# Imputation from SNP chip to sequence: a case study in a Chinese indigenous chicken population

**DOI:** 10.1186/s40104-018-0241-5

**Published:** 2018-03-21

**Authors:** Shaopan Ye, Xiaolong Yuan, Xiran Lin, Ning Gao, Yuanyu Luo, Zanmou Chen, Jiaqi Li, Xiquan Zhang, Zhe Zhang

**Affiliations:** Guangdong Provincial Key Lab of Agro-Animal Genomics and Molecular Breeding, National Engineering Research Centre for Breeding Swine Industry, College of Animal Science, South China Agricultural University, Guangzhou, Guangdong China

**Keywords:** Chickens, Imputation, Re-sequencing, SNP

## Abstract

**Background:**

Genome-wide association studies and genomic predictions are thought to be optimized by using whole-genome sequence (WGS) data. However, sequencing thousands of individuals of interest is expensive. Imputation from SNP panels to WGS data is an attractive and less expensive approach to obtain WGS data. The aims of this study were to investigate the accuracy of imputation and to provide insight into the design and execution of genotype imputation.

**Results:**

We genotyped 450 chickens with a 600 K SNP array, and sequenced 24 key individuals by whole genome re-sequencing. Accuracy of imputation from putative 60 K and 600 K array data to WGS data was 0.620 and 0.812 for Beagle, and 0.810 and 0.914 for FImpute, respectively. By increasing the sequencing cost from 24X to 144X, the imputation accuracy increased from 0.525 to 0.698 for Beagle and from 0.654 to 0.823 for FImpute. With fixed sequence depth (12X), increasing the number of sequenced animals from 1 to 24, improved accuracy from 0.421 to 0.897 for FImpute and from 0.396 to 0.777 for Beagle. Using optimally selected key individuals resulted in a higher imputation accuracy compared with using randomly selected individuals as a reference population for re-sequencing. With fixed reference population size (24), imputation accuracy increased from 0.654 to 0.875 for FImpute and from 0.512 to 0.762 for Beagle as the sequencing depth increased from 1X to 12X. With a given total cost of genotyping, accuracy increased with the size of the reference population for FImpute, but the pattern was not valid for Beagle, which showed the highest accuracy at six fold coverage for the scenarios used in this study.

**Conclusions:**

In conclusion, we comprehensively investigated the impacts of several key factors on genotype imputation. Generally, increasing sequencing cost gave a higher imputation accuracy. But with a fixed sequencing cost, the optimal imputation enhance the performance of WGP and GWAS. An optimal imputation strategy should take size of reference population, imputation algorithms, marker density, and population structure of the target population and methods to select key individuals into consideration comprehensively. This work sheds additional light on how to design and execute genotype imputation for livestock populations.

**Electronic supplementary material:**

The online version of this article (10.1186/s40104-018-0241-5) contains supplementary material, which is available to authorized users.

## Background

Genotype imputation [1] has become a common protocol of obtaining more genotypes at low cost by imputing from low to high density single nucleotide polymorphism (SNP) markers and even whole-genome sequence (WGS) SNP markers. It benefits whole genome studies, such as whole genome prediction (WGP) [2] and genome-wide association studies (GWAS) [3]. Both WGP and GWAS are used for genetic dissection and improvement of complex traits, based on the assumption of strong linkage disequilibrium (LD) between putative quantitative trait loci (QTL) and SNP markers. However, the estimated LD between SNPs rapidly decays with marker distance [4]. Obtaining higher density SNPs or whole genome sequence would enhance the performance of WGP and GWAS. But the existing genotyping arrays used in WGP and GWAS studies represent only a limited repertoire of sequence variation. Furthermore, it is known that a proportion of unexplained genetic components of complex traits (termed ‘missing heritability’ [5]) can be captured by rare variations. The number of detected rare variations detected can be increased by genotype imputation, although the imputation accuracy is usually low at rare sites [6]. Therefore, researchers are obliged to pursue high-density genotypes in these studies.

The availability of next generation sequencing (NGS) techniques has made it possible to obtain WGS SNP markers at a reasonable cost. However, sequencing thousands of individuals of interest is still too costly for routine implementation in livestock breeding programs. Recently, many studies have recommended the imputation of low-density SNP markers to WGS SNP markers. Genotype imputation is a widely used method that utilizes LD knowledge from haplotypes in a known reference panel to predict genotypes at missing or un-genotyped markers.

Genotype imputation had been implemented in human [7], cow [8], horse [9], dog [10], and chicken [11] and has identified many novel associated SNPs and QTLs. Using the comprehensive reference panels provided by the 1000 Human Genomes and 1000 Bull Genomes consortia, imputed whole genome-level SNPs have also recently became more common in human and bovine genomic studies [12–17]. For example, using imputed WGS data, Kelemen et al. [13] identified three novel risk SNPs associated with human mucinous ovarian carcinomas: rs752590 at 2q13 (*P* = 3.3 × 10^− 8^), rs711830 at 2q31.1 (*P* = 7.5 × 10^− 12^) and rs688187 at 19q13.2 (*P* = 6.8 × 10^− 13^). Pausch et al. [16] detected 12 new QTLs associated with mammary gland morphology in a German Fleckvieh cattle population.

Many factors affect the accuracy of genotype imputation, such as imputation algorithms [11], genetic relationship between reference and validation populations [18], the size of the reference population [8, 18], sequencing depth [19], and SNP density of the target panel [20]. With a layer chicken population, Ni et al. [11] evaluated performance of FImpute, Minimac and IMPUTE2 by imputing Affymetrix® Axiom® high-density SNP (600 K) data to WGS data, and FImpute was reported to be slightly worse than Minimac and IMPUTE2 in terms of genotype correlation. Pausch et al. [18] analyzed the impact of reference population on imputation accuracy when imputing from low-density to high-density SNPs in a Fleckvieh cattle population, and an increased imputation accuracy was observed as the reference population size and the relatedness between reference and target populations increased. Similar results were reported by van Binsbergen et al. [8]. Using simulated bovine sequence data at a given total cost, VanRaden et al. [19] found that sequencing more individuals at a low read depth could give a high accuracy of genotype imputation. Using a multi-breed sheep population genotyped with three SNP panels: 5 K, 50 K and 600 K SNPs, Ventura et al. [20] found that imputation accuracy could be improved by two-step imputation. Although the effect of each factor on the performance of genotype imputation has been reported, multi-factorial effects are still poorly understood and comprehensive and systematic investigations with real data are rarely reported.

In this study, we genotyped a Chinese indigenous chicken population using a chicken 600 K SNP chip, and we sequenced 24 selected key individuals. We systematically investigated the impacts of reference population size, key individual selection strategies, imputation algorithms, marker density of the target panel, sequencing depth, and the total cost of genotyping on the accuracy of genotype imputation when imputing array data to sequence data. Our results provide insight into the designing and executing of genotype imputation.

## Methods

### Population

The chicken population used in this study was derived from a Chinese indigenous breed and maintained for 25 generations by Wens Nanfang Poultry Breeding Co. Ltd. (Xinxing, P.R. China). The population consisting of 1,600 birds (800 males, 800 females), was the 3^rd^ batch of the 25^th^ generation of this chicken population. These birds came from a mixture of full sib and half sib families with the mating of 30 males and 360 females from the 24^th^ generation. After hatching, all birds were maintained in a closed building under controlled environmental conditions and provided with a standard diet till the end of 4 wk of age. Then they were randomly assigned to six pens by gender (three for male, and three for female) for growth performance testing from 5 to 13 wk of age. They received food and water ad libitum in all stages. Finally, slaughter was performed at 91 d of age and carcass traits recorded. In total, 1,338 birds (721 males, 617 females) were systematically phenotyped for further study.

### Genotyping by SNP chip

A total of 450 birds were selected for genotyping. These birds were 15 sires and 435 male offsprings. The average sire family size was 13.5 which ranged from 7 to 23. Genomic DNA of 450 individuals was extracted from blood samples using the NRBC Blood DNA Kit (Omega Bio-Tek, Norcross, GA, USA) according to the manufacturer’s instructions. DNA concentrations of samples were quantified and genotyped using the 600 K Affymetrix® Axiom® high-density genotyping array [21]. This SNP chip contains 580,961 SNP probes across 28 autosomes, two linkage groups (LGE64 and LGE22C19W28_E50C23), and two sex chromosomes. Genotyping was performed by Shanghai Biotechnology Corporation (Shanghai, China). Quality control criteria for SNP chip data were minor allele frequency (MAF) > 0.005, and individual genotyping call rate > 95%. Finally, 468,020 SNPs and 450 birds were used to build for G matrix.

### Key individual selection

The key individuals for re-sequencing were selected by maximizing the expected genetic relationship (REL), as described in detail by Druet and Hayes (2014) [22]. We utilized **G** matrix to replace the **A** matrix (pedigree-based genetic relationship matrix), to maximize the expected genetic relationship between key individuals and the remaining population, while maximizing the proportion of unique genomes sequenced in the population. Following previous studies [23, 24], the **G** matrix in this study was defined as $\mathbf{G}=\frac{\mathbf{M}{\mathbf{M}}^T}{\sum \limits_{i=1}^m{p}_i\left(1-{p}_i\right)}$, where **M** was an adjusted marker genotype matrix including *m* SNPs in columns and *n* individuals in rows. Here, the genotypes were coded as 0, 1, and 2 representing the copy number of the second allele, and then adjusted by 2*p*_*i*_ in each column, where *p*_*i*_ was the allele frequency of the second allele at the *i*th locus in the base population. Because the use of different allele frequencies *p*_*i*_ did not affect the accuracy of prediction [25–27], we used *p*_*i*_ = 0.5 for all SNPs to build all genomic relationship matrices, as in our previous studies [28].

### Whole-genome re-sequencing and variant calling

Key individuals selected from the previous procedures were re-sequenced with 150 bp paired-end reads on the Illumina HiSeq 3000 platform. The sequencing was performed by RiboBio Co., Ltd. (Guangzhou, China). Briefly, the initial quality of raw reads was checked using FastQC [29], with a Phred score of 20 as the minimum to remove the adaptor polluted reads and multiple *N* reads (where *N* > 10% of one read) to produce clean reads. Then the clean reads were aligned to the chicken reference genome (galGal4) using the Burrows-Wheeler Alignment tool (BWA, version 0.7.12) [30] with default parameters. The SAM files generated from BWA were converted to BAM files by SAMtools (version 1.2) [31]. After that, potential PCR duplicates were removed by the MarkDuplicates utility in Picard release 1.119 [32]. Lastly, the BAM files were further processed with the UnifiedGenotyper utility of GATK (version 3.5) [33] to call the SNPs with multi-sample approaches and to filter out false positive variants with the following parameters: variant confidence score (QUAL) ≥ 50, QualByDepth (QD) ≥ 2.0, total depth of coverage (DP) ≥3, FisherStrand (FS) < 60, and to remove SNPs clusters [34, 35]. After filtering, the remaining VCF file with GT field data was converted to a Beagle (v3) genotypes file by Beagle utilities for further analysis [36].

### Validation of variant detection

For each key individual, the concordances of SNPs called by GATK were evaluated by SNP genotypes obtained from the 600 K array. As proposed by Baes et al. [37], four measures of concordances, SNP concordance (SC), genotype concordance (GC), non-reference sensitivity (NRS), and non-reference discrepancy (NRD), were used to assess the concordance between WGS data and 600 K data. The four measures were evaluated using 600 K data set as the total sample positions in the WGS data. For each key individual, SC was the proportion of genotypes that were non-missing genotypes in the WGS data over all non-missing genotypes in the 600 K data. GC was the proportion of array-derived genotypes that were the same as the sequence-derived genotypes over all non-missing genotypes of the sequence-derived genotypes. NRS was the proportion of genotypes that have at least one non-reference allele (NRA) in both WGS data and 600 K data over the total number of genotypes detected to have at least one NRA in 600 K data. NRD was the proportion of genotypes in which sequence-derived genotypes were different from array-derived genotypes over the total sample positions.

### Genotype imputation

To investigate the influences of the imputation algorithms, SNP density of the target panel, the number of sequenced individuals, selection strategies, sequencing depth, and the total cost of genotyping were assessed on the imputation accuracy from SNP chip data to WGS data. Four scenarios were considered in genotype imputation section (denoted as S1, S2, S3, and S4).

Scenario one (S1) was designed to investigate the effect of target panel density on imputation accuracy from SNP chip data to WGS data. S1 contained three imputation sections: direct imputation from low-density chip (60 K) to WGS data, direct imputation from high-density chip (600 K) to WGS data, and indirect imputation from 60 K to 600 K data and then from 600 K to WGS data (two-step imputation approach). The supposed 60 K chip data was generated by sampling the first SNP in each bin of adjacent 10 SNPs from the 600 K SNP chip. Scenario two (S2) was designed to study the effects of the number of sequenced individuals and selection strategies on imputation accuracy from 600 K to WGS data. S2 was performed by adding sequenced individuals to the reference population one by one with optimized or random rank, respectively. In S2, the optimized rank was defined as the priority queue of key individuals determined by REL. Also we fixed the sequence read depths (X) of sequenced individuals as 12X. Scenario three (S3) was designed to study the effects of sequenced depths on imputation accuracy. We fixed the number of sequenced individuals (N) as 24 and changed the sequence read depths from 1 to 12X. Scenario four (S4) was designed to study the effects of different WGS data costs on imputation accuracy and to assess optimal sequencing strategies. The total cost of WGS data was defined as that the number of sequenced individuals multiplied by the sequence depth of each sequenced individual (Table 1). The optimal sequencing strategy was defined as the combination of sequencing depth and number of sequenced individuals, which gives the highest imputation accuracy. In this study, different sizes of WGS datasets were randomly sampled from complete WGS data of 24 sequenced individuals. We defined 1X to be made up of 7,000,000 reads, which consisted of 1,050,000,000 bases. We used a Bioconductor package (ShortRead [38]) to read and write the clean fastq file of sequenced individuals. After reading the fastq file, we randomly selected reads to make up different depth fastq files of sequenced individuals. These different depths of sequencing data were aligned on the reference genome and variants were called by the above procedures. We did not control genotype quality to retain more SNPs for further analyses.

**Table 1:** Sequencing strategies used for genotype imputation

Total X	Different sequencing scenarios with fixed cost (X × N)						
24	1 × 24	2 × 12	3 × 8	4 × 6	6 × 4	8 × 3	2 × 12
36	2 × 18	3 × 12	4 × 9	6 × 6	9 × 4	12 × 3	18 × 2
72	3 × 24	4 × 18	6 × 12	8 × 9	9 × 8	12 × 6	18 × 4
96	4 × 24	6 × 16	8 × 12	12 × 8	16 × 6		
144	6 × 24	8 × 18	12 × 12	16 × 9	18 × 8		

*Total X* total cost of genotyping, *X × N* sequenced depth (X) times the number of sequenced animals (N)

Imputations were executed by FImpute (Version 2.2) and Beagle (Version 3.3.2) with default parameter settings. FImpute was based on an overlapping sliding window method in which information from close relatives (long haplotype match) was first utilized, and information from distant relatives was subsequently used by shortening the window size [39]. Beagle used a hidden Markov model and a localized haplotype clustering method to infer genotypes as described by Browning et al. [36].

The quality control criteria of SNP chip data were minor allele frequency (MAF) > 0.005, individual genotyping call rate > 95%, and SNP genotyping call rate > 97%. Finally, 462,092 SNPs and 444 birds remained for further investigation. We randomly masked 2% of SNPs from the 600 K SNP panel of each individual to be imputed, and then compared the imputed genotypes with array-derived genotypes to calculate the imputation accuracy. The imputation accuracy was defined as the average genotype concordance from five replicates. To improve computational efficiency, chromosomes 1, 3, 6, and 28 (chr1, chr3, chr6, and chr28) were selected.

## Results

### Selection of key individuals and whole genome re-sequencing

**Fig. 1:**
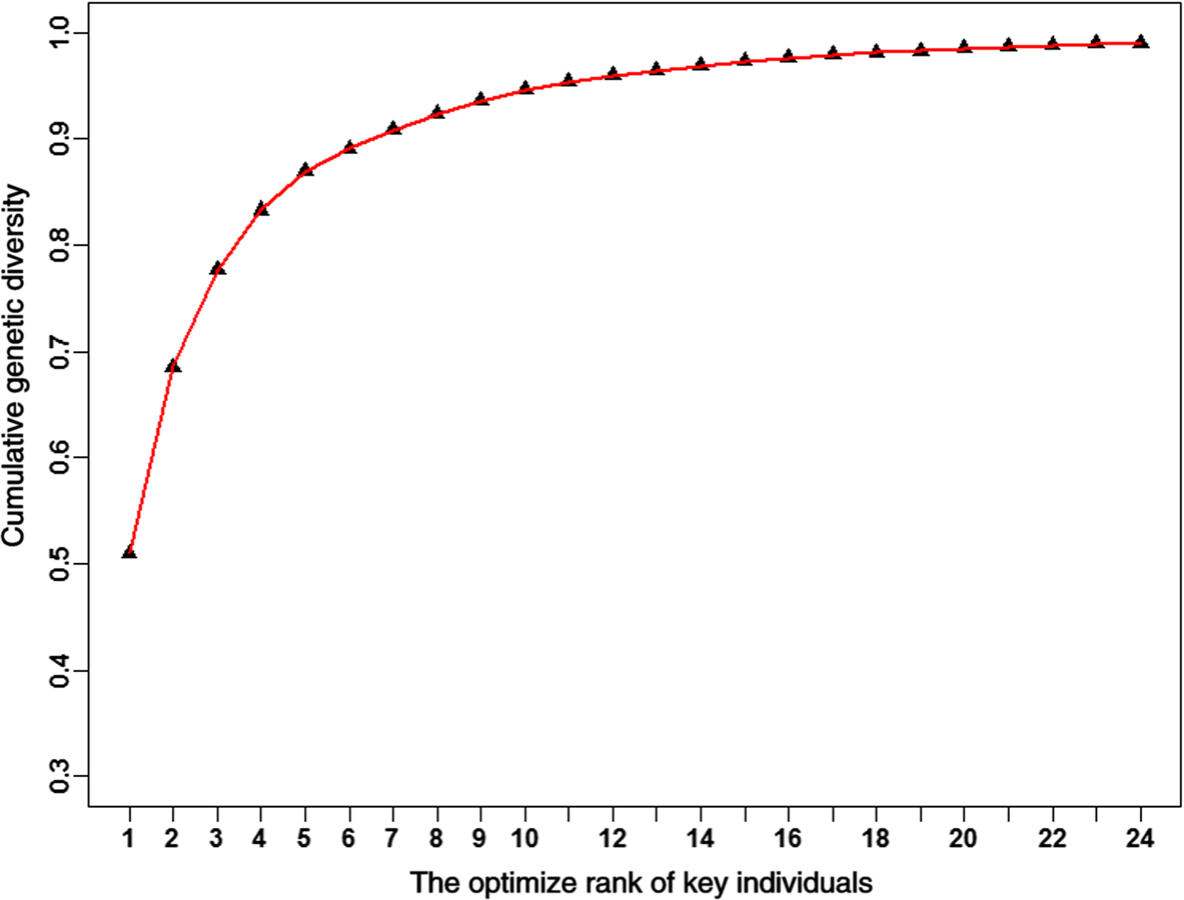
The cumulative genetic diversity of selected key individuals was estimated by adding animals with the optimize rank of 24 key individuals one by one. The cumulative genetic diversity means the proportion of the entire chicken population

We calculated the marker-based genetic relationship matrix (G matrix) of 450 genotyped chickens with 600 K array data. All genotyped individuals were ordered by maximizing the expected genetic relationship (REL), using G matrix, between the group of sequenced birds and the whole population. The 24 birds that showed the highest values were selected as key individuals. These 24 birds from 21 sire family were three male parents (ID: 7, 8, 9) and 21 male offspring, and their relatedness is shown in Additional file 1: Table S1. The cumulative genetic diversity of selected key individuals increased as the numbers of selected key individuals increased, but the rate of increase gradually slowed (Fig. 1). On average, 98.99% of genetic diversity in the entire chicken population was covered by variations in the genomes of these 24 key birds (Fig. 1). After quality control, 450.6 Gb of clean data were generated from the 24 birds, and 428.2 Gb could be mapped to the chicken reference genome (galGal4). Among all key individuals, the average uniquely mapped efficiencies of mapped reads were 95% (from 87% to 97%), and the average sequenced depths were 14.62 (from 12.87 to 17.11). More details of the 24 sequenced chickens are shown in Table 2 and Additional file 1: Table S2.

**Table 2:** Summary of the key individuals for re-sequencing

Animal ID	Clean Reads	Mapped reads rate	Depth of coverage	Uniquely mapped reads rate	SC	GC	NRS	NRD
1	90,735,760	0.951	15.81	0.942	0.988	0.979	0.972	0.042
2	117,996,876	0.964	12.87	0.959	0.998	0.928	0.947	0.128
3	105,660,610	0.938	16.21	0.928	0.975	0.979	0.961	0.040
4	125,426,182	0.953	17.11	0.947	0.974	0.976	0.954	0.047
5	111,564,528	0.956	13.60	0.951	0.997	0.985	0.989	0.029
6	127,019,556	0.960	14.68	0.955	0.998	0.957	0.968	0.079
7	99,058,934	0.873	15.11	0.864	0.990	0.976	0.974	0.046
8	130,604,192	0.961	15.19	0.956	0.953	0.991	0.951	0.018
9	141,700,352	0.961	16.25	0.956	0.999	0.991	0.995	0.017
10	141,847,268	0.965	14.39	0.960	0.999	0.991	0.995	0.018
11	115,053,394	0.958	13.92	0.953	0.997	0.986	0.989	0.026
12	141,220,480	0.965	14.41	0.961	0.999	0.992	0.995	0.016
13	126,408,732	0.959	14.44	0.951	0.997	0.953	0.964	0.087
14	137,853,286	0.963	14.79	0.958	0.998	0.989	0.993	0.022
15	124,123,884	0.961	13.92	0.955	0.998	0.987	0.991	0.026
16	134,906,464	0.970	13.88	0.965	0.999	0.990	0.995	0.020
17	137,609,612	0.957	13.77	0.950	0.998	0.988	0.992	0.024
18	140,592,166	0.954	14.53	0.946	0.998	0.990	0.993	0.020
19	120,575,426	0.955	14.53	0.949	0.996	0.986	0.988	0.028
20	131,305,824	0.962	14.29	0.957	0.998	0.980	0.988	0.038
21	140,828,990	0.964	15.43	0.959	0.997	0.989	0.992	0.020
22	134,838,280	0.965	14.33	0.960	0.998	0.966	0.977	0.064
23	115,132,512	0.951	13.51	0.944	0.997	0.988	0.989	0.024
24	112,104,986	0.953	13.84	0.947	0.998	0.989	0.990	0.022

*SC* SNP concordance, *GC* Genotype concordance, *NRS* Non-reference sensitivity, *NRD* Non-reference discrepancy

### Variant detection and validation

For the 24 key individuals, 13,818,577 SNPs were called by GATK. After quality control, 11,645,758 SNPs remained for further analysis (Additional file 1: Table S3). Concordances of WGS data and 600 K data were compared for SNPs represented in both WGS and array data (Table 2). On average, concordance between two panels was 99.3%, 98.0%, 98.1%, and 3.75% for SNP concordance (SC), genotype concordance (GC), non-reference sensitivity (NRS), and non-reference discrepancy (NRD), respectively (Table 2). The high SC, GC and NRS values but low NRD value indicated that the accuracy of genotype calls was very high.

### Variable target panel SNP density in genotype imputation

**Fig. 2:**
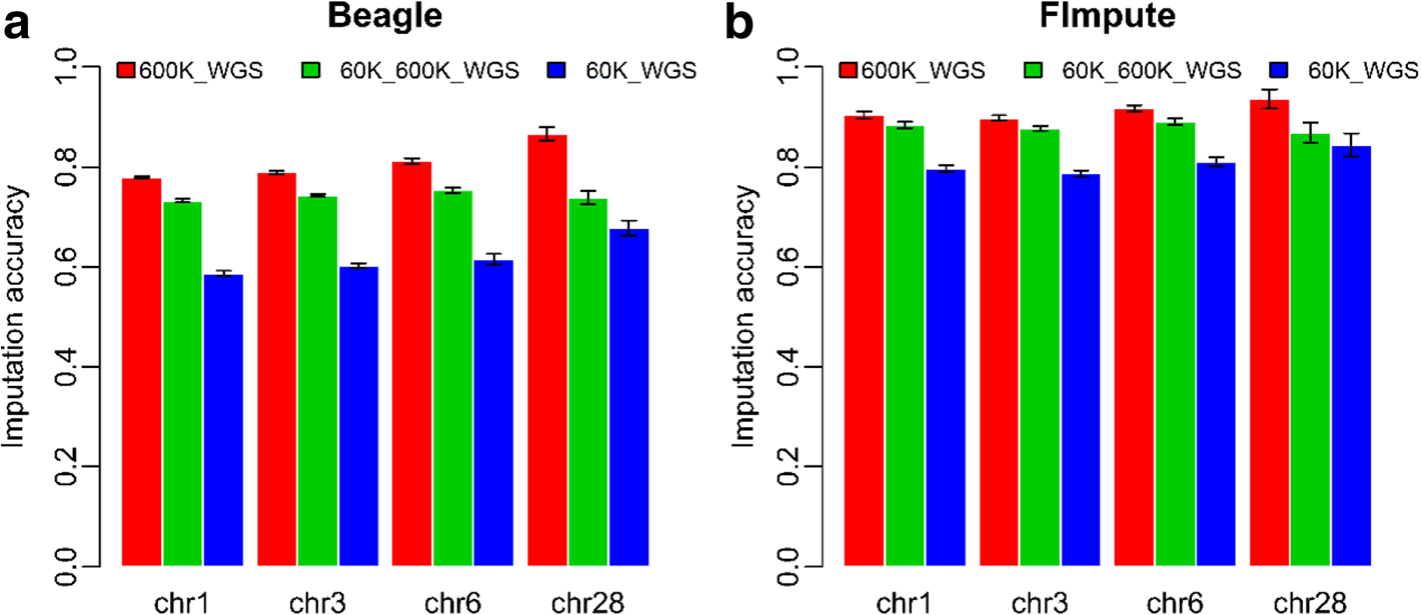
Average imputation accuracy of the direct imputation and two-step imputation obtained with FImpute and Beagle against four chromosomes (chr1, chr3, chr6 and chr28) among 5 replications. 60K_WGS was the direct imputation from 60 K to WGS data. 60K_600K_WGS was the two-step imputation from 60 K to 600 K data and then to WGS data. 600K_WGS was the direct imputation from 600 K to WGS data. The imputation accuracies were the genotype concordance between the true and imputed genotypes

Average imputation accuracies (five replicates) of SNP panels with different densities to WGS data for four chromosomes (chr1, chr3, chr6, and chr28) are shown in Fig. 2. For direct imputation from 600 K to WGS data, the average imputation accuracy for the four chromosomes was 0.812 for Beagle (ranging from 0.780 to 0.867) and 0.914 for FImpute (ranging from 0.898 to 0.936). For direct imputation from 60 K to WGS data, the average imputation accuracy for the four chromosomes was 0.620 for Beagle (ranging from 0.587 to 0.667) and 0.810 for FImpute (ranging from 0.797 to 0.844). Furthermore, for the two-step imputation approach from 60 K to 600 K data and then to WGS data, the average imputation accuracy for the four chromosomes was 0.742 for Beagle (ranging from 0.732 to 0.753) and 0.880 for FImpute (ranging from 0.869 to 0.891). These values were higher than those for direct imputation from 60 K to WGS data but lower than those for direct imputation from 600 K to WGS data. Compared with direct imputation from 600 K to WGS data, the imputation accuracy of the two-step imputation approach from 60 K to WGS data lost less than 4% for FImpute and 9% for Beagle, but reduced the genotyping cost for the target panel by 90%.

### Variable sequence depth for a fixed number of sequenced individuals

**Fig. 3:**
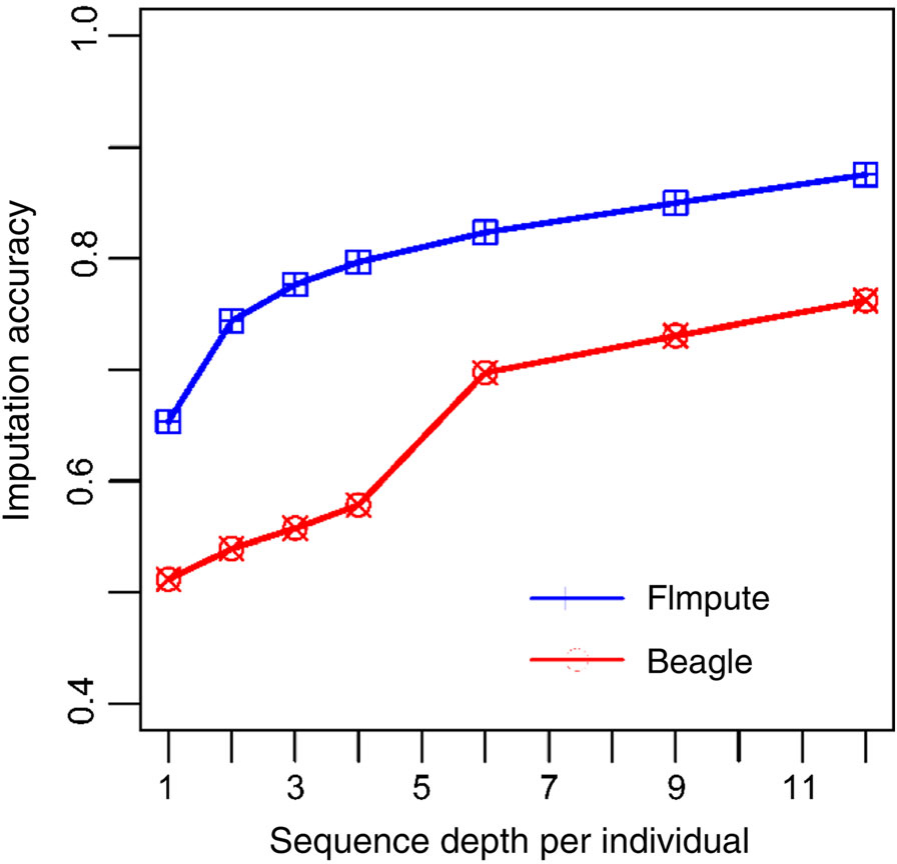
Average imputation accuracies of different X with fixed N (N = 24) obtained with FImpute and Beagle against four chromosomes (chr1, chr3, chr6, and chr28). The imputation accuracies were the genotype concordance between the true and imputed genotypes

With all 24 key individuals as a reference, imputation accuracies under different sequencing depths are shown in Fig. 3. An increasing imputation accuracy was observed as the sequence depth increased. Beyond a sequencing depth of 6X, the increase in accuracy slowed down for both FImpute and Beagle (Fig. 3). For example, when the sequence depth was increased from 6X to 12X, the total cost of sequencing increased by 100%, but the imputation accuracy only increased by 6.0% and 9.0% for FImpute and Beagle, respectively. Further details for each chromosome can be seen in Additional file 2: Figure S1. These results indicate that imputation accuracy increased as sequence depth increased, but the change was not linear.

### Variable number of sequenced individuals with fixed sequence depth

**Fig. 4:**
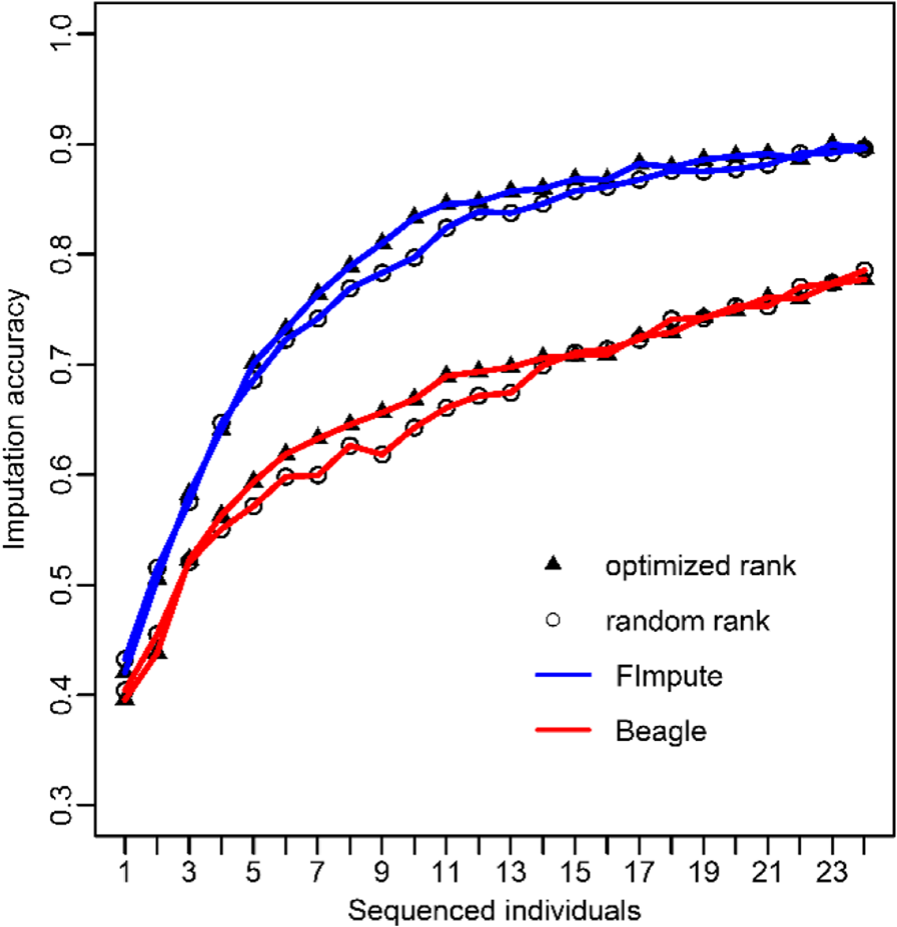
Average imputation accuracies of 12 X with different N (1~ 24) obtained with FImpute and Beagle from 5 replications on chromosome 6. The imputation accuracies were the genotype concordance between the true and imputed genotypes

To investigate the influences of different selection strategies and reference size on imputation accuracy, we randomly selected different numbers of individuals from these 24 key individuals using random rank and optimized rank (Fig. 4). We found that a greater number of sequenced individuals result in a higher imputation accuracy at fixed sequence depths (X = 12X). In addition, there was no obvious difference in imputation accuracy between optimized and random rank when the size number were from 1 to 5. There was also no obvious difference when the reference size was 12 to 24 for FImpute, and 14 to 24 for Beagle (Fig. 4). However, when the reference size was 6 to 11 for FImpute and Beagle, the imputation accuracy of optimized rank was higher than that of random rank.

### Variable total sequence cost and the optimal sequencing strategy

**Fig. 5:**
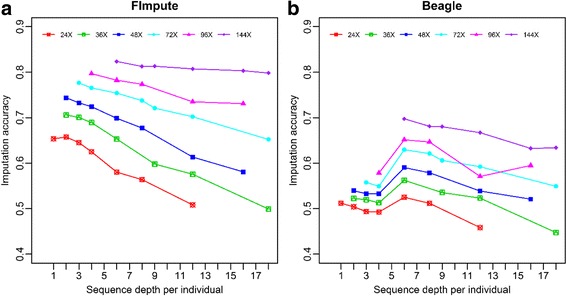
Average imputation accuracy of different total cost obtained with FImpute and Beagle against four chromosomes (chr1, chr3, chr6, and chr28) among 5 replications. A given total cost was defined as the number of sequencing individuals timed the sequence read depth of each individuals. The imputation accuracies were the genotype concordance between the true and imputed genotypes

The average imputation accuracies of FImpute and Beagle at different sequence depths for different numbers of sequenced individuals are shown in Fig. 5. Imputation accuracy increased with increasing total coverage depth (24X, 36X, 48X etc.), indicating that greater imputation accuracy might require higher sequencing cost. For FImpute, at the same total coverage depths, the imputation accuracy increased with the number of sequenced individuals. However, for Beagle, the imputation accuracy reached a maximum at 6X per sequenced individuals. For example, when the total coverage depth was 36X, the highest imputation accuracy was 0.523 at 6X per sequenced individual. When the total coverage depth was increased to 72X, the highest imputation accuracy was still at 6X per sequenced individual. Further details for each chromosome are presented in Additional file 2: Figure S2.

### Effect of minor allele frequency (MAF) on imputation accuracy

**Fig. 6:**
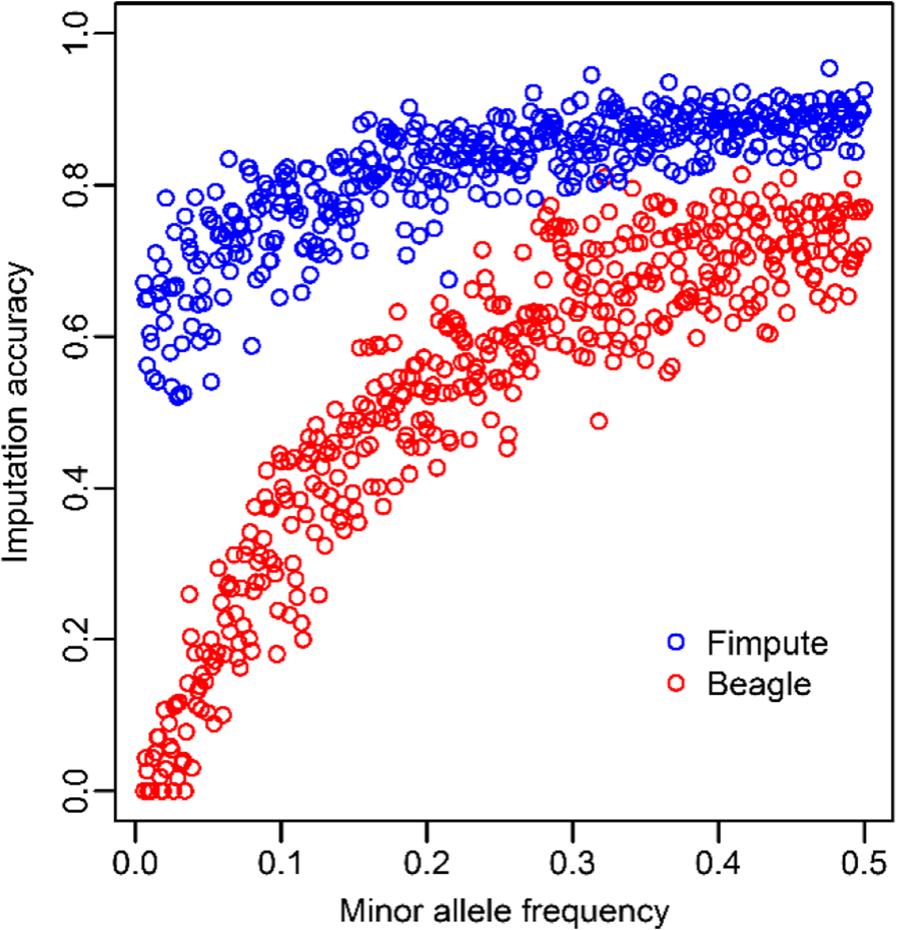
Average imputation accuracies of different software against minor allele frequency among 5 replications. SNPs were classified by their array-derived MAF

The average imputation accuracies of SNPs with different MAFs with FImpute and Beagle were calculated and are shown in Fig. 6. We found that the average imputation accuracy of FImpute was higher (0.822) than that of Beagle (0.533). Beagle performance was suboptimal for SNPs with a MAF smaller than 0.2. Imputation accuracies from FImpute were comparatively stable with different MAFs, but there was a small reduction when MAF was low (< 0.1).

## Discussion

### Imputation from SNP chip data to sequence data

In this study, we were specifically interested in influences of the size of the sequenced key individuals, selection strategies, imputation algorithms, marker density of the target panel, sequencing depth, and the total cost of genotyping on the accuracy of genotype imputation from the SNP chip data to WGS data. For all scenarios, genotype imputations were separately performed using FImpute and Beagle. The reference panels were built from 24 key sequenced individuals or subsets of them, as selected by the REL model. In this study, the number of SNPs was successfully raised more than 25 times from 600 K SNPs (462092) to WGS SNPs (11645758) (Additional file 1: Table S3) with a high imputation accuracy (0.812 for Beagle and 0.914 for FImpute) (Fig. 2). These high imputation accuracies are in agreement with previous reports on chickens and bovines [8–11, 40]. In another imputation study on chickens [11], the imputation accuracies of different programs were all more than 0.95 from 600 K data to WGS data. In bovines, the imputation accuracies from BovineHD (40,492 SNPs) bead chips to WGS data ranged from 0.77 to 0.83 for Beagle [8]. The main aim of genotype imputation is to improve the chip density for GWAS or WGP; however, imputation errors will affect the performance of GWAS or WGP [41]. One way to avoid imputation error is to improve imputation accuracy by using large reference size [8, 18], sequencing large depth [19]. Another way is to strictly control the quality of imputed WGS data [42]. Imputed WGS data has become more common in human and bovine genomic studies [12–17].

### Genotyping strategy for imputation

A key question arising from the sequencing strategy design was how to balance the number of sequenced individuals and the depth of sequence with a given genotyping cost. Our observations indicated that the accuracy of imputation continuously increased as the genotyping cost increased, whether it be by increasing the number of sequenced individuals with a fixed sequence depth or increasing sequence depth with a fixed number of sequenced animals (Fig. 3, Fig. 4). However, the rate of increase gradually slowed down for FImpute and a similar trend was found for Beagle when increasing sequence depth was more than 4X (Fig. 3). Similar results were reported by VanRaden et al. [19]. Therefore, an optimum genotyping strategy exists for imputation with the fixed genotyping cost.

With the fixed genotyping cost, imputation accuracy was related to imputation software, sequence depth, and the number of sequenced animals. Using Beagle, an optimal sequence depth around 6X was clearly observed in our results (Fig. 5), which suggested that an optimal sequence depth existed for a population when a pedigree was not available. Also, Druet et al. [13] found that the optimum strategy was to sequence 75 individuals at eightfold coverage using Beagle if the total sequencing effort was constrained to 600X. However, we observed that the lower sequencing depth with more sequenced individuals granted a higher imputation accuracy for FImpute (Fig. 5). Similar results have been reported for findhapV4, which also uses family-based methods for imputation [11]. Also, more variants were detected by sequencing as many individuals as possible at a low fold coverage (Additional file 1: Table S4). A similar result was found by Le and Durbin [43] indicating that using family-based methods for imputation and sequencing as many individuals as possible at a low-fold coverage not only capture more SNPs but also improve imputation accuracy for a given total cost of genotyping.

In this study, the initial density of the SNP panel was considerably decreased (90% in this study, 60 K vs. 600 K), while the loss of imputation accuracy was only less than 4% for FImpute and 9% for Beagle using a two-step imputation approach (Fig. 2). This result indicated that, in practice, the genotyping cost for a large population could be largely reduced by genotype imputation with only a tiny loss in imputation accuracy if most animals in the nucleus of a breeding population were related. Additionally, our results indicate that genotyping cost can be decreased by genotyping a population with a customer designed low-density panel (60 K) rather than the high density panel (600 K). But no money would be saved if most animals were in the nucleus of a breeding population were distantly related to each other because the number of key individuals genotyped with the high density chip was a large proportion of the nucleus breeding population. The cost calculations of genotyping were based on the current price of sequencing and genotyping arrays.

### Key individual selection

Key individual selection was effective for genotype imputation. We selected 24 representative chickens for re-sequencing using the REL model. The sequenced individuals were added to the reference population one by one using optimized or random rank. We predicted that the advantage of optimized rank over random rank would be observed at the middle stage of this comparison. This was because the individuals used for random rank were the same 24 key selected chickens with a random resampling rank, and these 24 key individuals were the most representative ones in the population. Our observation met well with our prediction (Fig. 4). In addition, key individual selection can maximize correct imputation of the variant by maximizing genetic variation. Similar results have been previously reported [18, 44]. However, Yu et al. [45] found that animals with the closest average relationship or contribution to the target population gave the lowest accuracy imputation, in some cases worse than random selection.

### Imputation methods

We compared the performance of Beagle and FImpute for all scenarios. Generally, FImpute outperformed Beagle in the present population. The superiority of FImpute over Beagle was also observed in previous studies [9, 39, 46, 47]. The advantage of FImpute over Beagle observed in this study might be explained by the fact that FImpute can capture similar haplotypes between close relatives via a pedigree-based imputation method, while Beagle cannot find the most likely haplotype based solely on the known genotypes of limited individuals using population-based imputation methods. However, the differential performance between the two imputation methods would be diluted by using a large reference panel, and this has been reported in cattle (*n* = 1652) [48]. In this study, the difference between FImpute and Beagle results from the use of a small reference population and family structure. Moreover, FImpute analysis was more rapid compared with Beagle (Table 3).

**Table 3:** Summary of imputation from 600 K to WGS data

Chr.	SNP # in sequence	SNP # in chip	SNP # for validation	Total time-consuming, s	
				Beagle	FImpute
1	3,177,578	81,074	1,621	403,680	3,066
3	1,694,589	45,917	918	232,456	1,773
6	622,557	17,762	355	90,281	727
28	74,114	3,866	77	1,788	111

There are three versions of Beagle (Beagle v.3.3.2, Beagle v.4.0, and Beagle v.4.1). We compared the imputation accuracy with Beagle and FImpute with or without pedigree in these recent versions (Additional file 2: Figure S3). The imputation accuracy was the genotype concordance between the true and imputed genotypes. We found that the imputation accuracy of Beagle v.4.0 was similar to that of FImpute, whether using the pedigree or not. Because Beagle v.4.0 applied a new method for identity by descent (IBD) segment detection (Refined IBD) to improve methods for phasing and genotype imputation [49]. Refined IBD was similar to the method of FImpute in capturing similarity haplotypes between close relatives. And the imputation accuracy of Beagle v.4.1 was less than that of Beagle v.4.0 without pedigree because Beagle v.4.1 has a very fast genotype imputation algorithm for genotype imputation with millions of reference samples [50]. The imputation accuracy of FImpute without pedigree was better than that of FImpute with pedigree. This may be caused by Mendelian errors with the pedigree. The imputation accuracy is similar for Beagle v.4.0 with or without the pedigree. This was the result of the family structure of this population for IBD segment detection.

Overall, the population size, structure, computational efficiency, and other key factors should be comprehensively considered to select an appropriate imputation method or software.

### Length of chromosome

Imputation accuracy from SNP chip data to WGS was similar among chr1, chr3, and chr6, but not chr28 (Fig. 2). This might be caused by standard error in chr28. Only 3886 SNPs on chr28 were captured by the 600 K array, which was less than that of chr1 (81,074), chr3 (45,917), and chr6 (17,762). Hence, using 2% masked true genotypes (77 SNPs) to evaluate the imputation accuracy would produce larger statistical standard error (Fig. 2). For example, with Beagle, in the imputation from 600 K to WGS data, we found that the standard deviation of imputation accuracies for chr28 was 2.71, 3.80 and 3.17-fold larger than for chr1, chr3, and chr6, respectively (Fig. 2). Furthermore, with FImpute, it was 6.50, 4.33 and 2.17-fold larger than for chr1, chr3 and chr6, respectively (Fig. 2). However, for two-step imputation from 60 K to WGS data, the imputation accuracy for chr28 was the lowest compared with the other chromosomes (chr1, chr3, and chr6) (Fig. 2). This might be because the number of SNPs per centi-Morgan influenced imputation error rate more than for the other chromosomes (chr1, chr3, and chr6) using two-step imputation [51]. Because of higher recombination rates in the microchromosomes, their LD and haplotype sharing were significantly lower compared to the macrochromosomes [52]. Moreover, a slight decrease in imputation accuracy for the shorter chromosomes was observed by Sun et al. [53] in Angus cattle. The slightly lower accuracy on the shorter chromosomes can be explained by the reduced accuracy at the beginning and end of the chromosome which would have a relatively larger effect for the short chromosomes. However, another study did not find a difference in imputation accuracy between chromosomes of different lengths [54]. In practice, the length of chromosomes does not need to be considered for genotype imputation.

### Minor allele frequency (MAF)

It has been suggested that SNPs with low allele frequency may play an important role in complex traits, and may have larger effects than the common SNPs in a population [55]. However, correctly imputing rare SNPs is still a challenge. In our study, the imputation accuracy decreased sharply with MAF < 0.2, especially for Beagle (Fig. 6). The lower imputation accuracy of low MAF SNPs was in agreement with other studies [56–59]. Lin et al. [59] showed with human data that the decline in imputation accuracy already started with MAFs < 0.15. Hickey et al. [58] and Hayes et al. [57] also reported a decline in imputation accuracy for MAFs < 0.1 in maize and sheep populations. Interestingly, for FImpute, the selection of the key animal to sequence appears to especially benefit imputation accuracy of low MAF SNPs.

### Validation of variant detection

The comparison of array-derived genotypes versus sequence-derived genotypes provides an objective quality measurement for NGS experiments and the variant calling pipeline. Our results revealed that both GC and NRS were very high, =ranging from 0.928 to 0.992 and from 0.947 to 0.995, respectively. But the average NRD of 24 key individuals was 0.037. Moreover, Pearson’s correlation coefficient between NRD and the depth coverage of 24 key individuals was − 0.18 (*P* = 0.41). These values were similar to those observed in 43 Fleckvieh cattle [60], and we found that low coverage (< 7×) had a negative effect on both of these parameters. These results are consistent with previous studies [37], indicating that the variant calling pipeline in this study was conducted correctly.

## Conclusions

In conclusion, we comprehensively investigated the impacts of several key factors on the outcome of genotype imputation. Generally, increasing sequencing cost gave a higher imputation accuracy. But at a fixed sequencing cost, the optimal imputation strategy should take sequencing depth and size of reference population, imputation algorithms, marker density, and population structure of the target population and method to select key individuals into consideration comprehensively. This work sheds additional light on how imputation algorithms, selection strategy for key individuals, and design of the sequencing plan influences accuracy of genotype imputation in livestock populations.

## Additional files

Additional file 1: **Table S1.** A pedigree-based genetic relationship matrix among the 24 key individuals. **Table S2** A summary of the sequencing and assembly of 24 key individuals. **Table S3** Number of identified variants in the chicken genome with GATK. **Table S4** The results of the SNP calling for chromosomes (chr1, chr3, chr6, and chr28) with different sequencing depth under the different total cost of genotyping, respectively. (XLSX 25 kb)

Additional file 2: **Figure S1.** Imputation accuracy in different total X obtained with FImpute and Beagle against 4 chromosomes (chr1, chr3, chr6, and chr28) among 5 replications, respectively. **Figure S2** Imputation accuracy in different X with fixed *N* (*N* = 24) obtained with FImpute and Beagle against 4 chromosomes (chr1, chr3, chr6, and chr28) among 5 replications, respectively. **Figure S3** The average imputation accuracy of direct imputation from 600 K to WGS data obtained with FImpute, Beagle v.3.3.2, Beagle v.4.0, and Beagle v.4.1 against four chromosomes (chr1, chr3, chr6 and chr28) among 5 replications. (DOCX 1755 kb)

## Abbreviations

DP: Total depth of coverage
FS: FisherStrand
GC: Genotype concordance
GWAS: Genome-wide association studies
IBD: Identity by descent
LD: Linkage disequilibrium
MAF: Minor allele frequency
N: The number of sequenced individuals
NGS: Next generation sequencing
NRD: Non-reference discrepancy
NRS: Non-reference sensitivity
QD: QualByDepth
QTLs: Quantitative trait locus
QUAL: Variant confidence score
SC: SNP Concordance
SCAU: South China Agricultural University
SNP: Single nucleotide polymorphism
WGP: Whole genome prediction
WGS: Whole-genome sequence
X: Sequence read depths
