# Supplementary material for: Imputation from SNP chip to sequence: a case study in a Chinese indigenous chicken population
Source: J Anim Sci Biotechnol. 2018 Mar 21;9:30. doi: 10.1186/s40104-018-0241-5 (PMC5861640; doi:10.1186/s40104-018-0241-5)
Supplement: Supplementary file 2 — Figure S1. Imputation accuracy in different total X obtained with FImpute and Beagle against 4 chromosomes (chr1, chr3, chr6, and chr28) among 5 replications, respectively. Figure S2 Imputation accuracy in different X with fixed N (N = 24) obtained with FImpute and Beagle against 4 chromosomes (chr1, chr3, chr6, and chr28) among 5 replications, respectively. Figure S3 The average imputation accuracy of direct imputation from 600 K to WGS data obtained with FImpute, Beagle v.3.3.2, Beagle v.4.0, and Beagle v.4.1 against four chromosomes (chr1, chr3, chr6 and chr28) among 5 replications. (DOCX 1755 kb) [file 40104_2018_241_MOESM2_ESM.docx]

***Fig. S1***


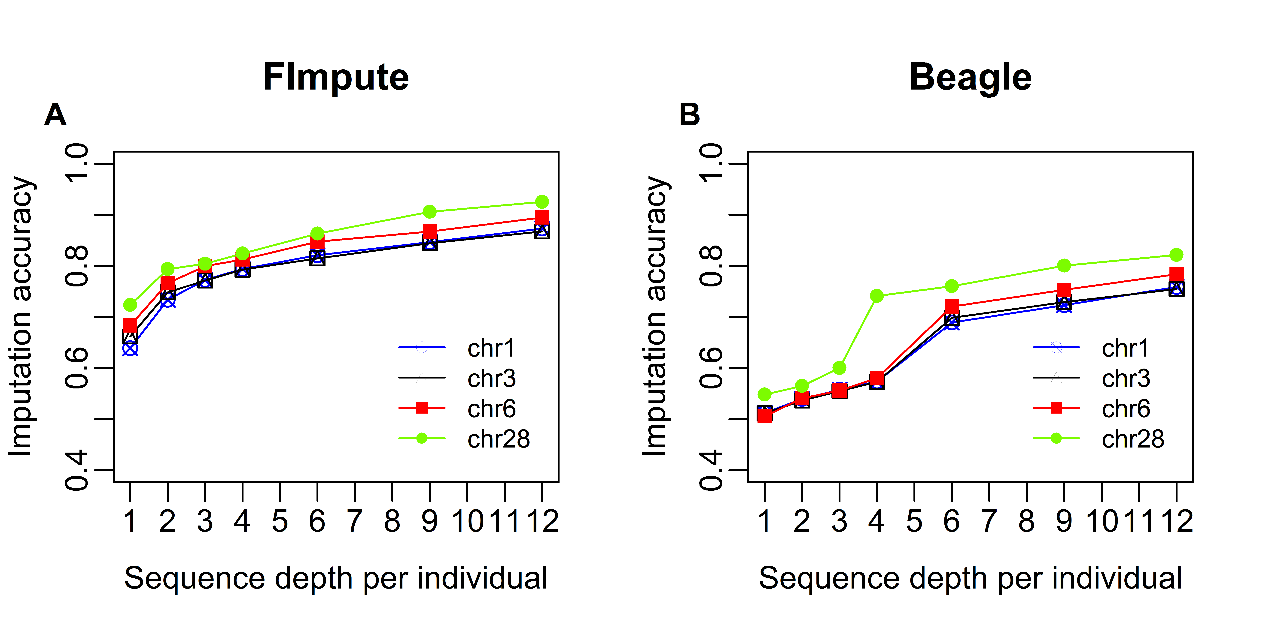


Imputation accuracy in different X with fixed N (N = 24) obtained with FImpute and Beagle against 4 chromosomes (chr1, chr3, chr6, and chr28) among 5 replications, respectively. The imputation accuracy were the genotype concordance between the true and imputed genotypes.

***Fig. S2***


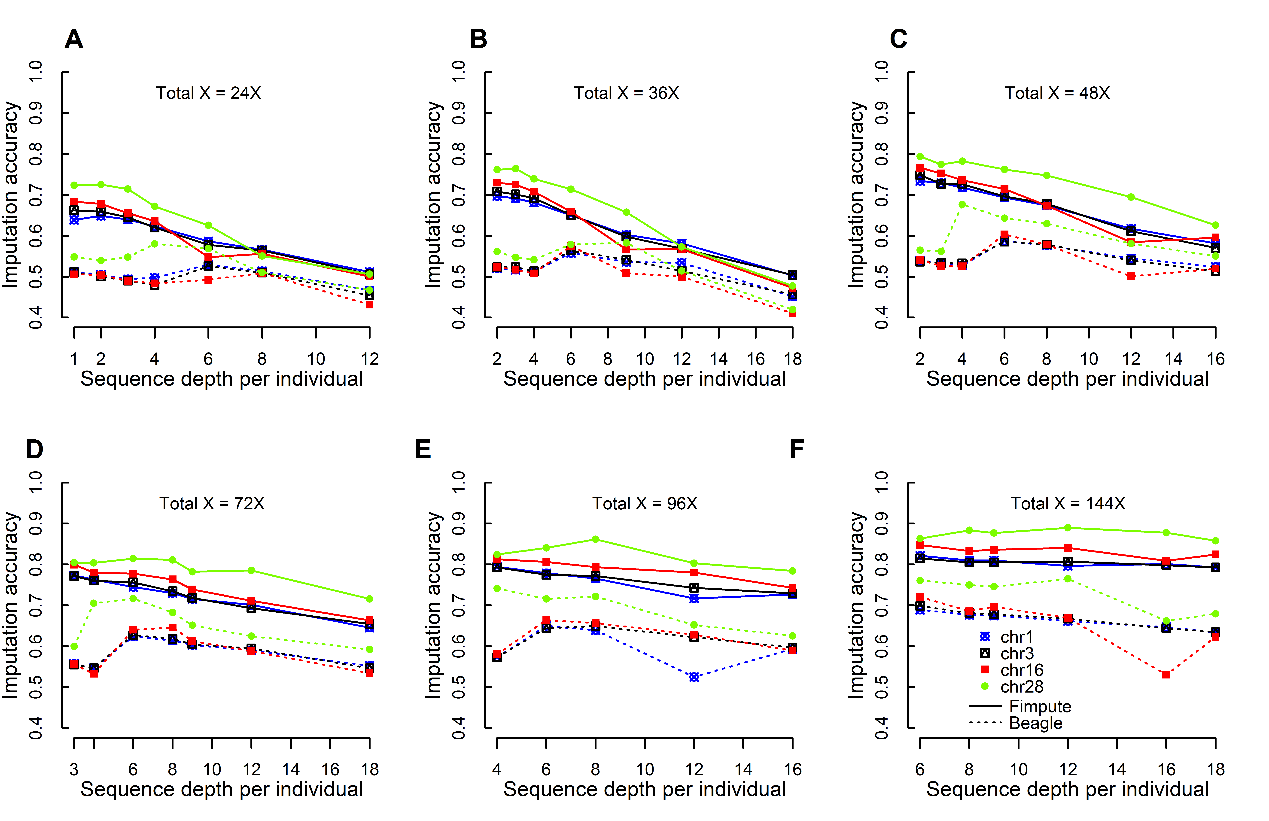


Imputation accuracy in different total X obtained with FImpute and Beagle against four chromosomes (chr1, chr3, chr6, and chr28) among 5 replications, respectively. A given total X based on the number of sequencing animals times sequence depth. The imputation accuracy were the genotype concordance between the true and imputed genotypes.

***Fig. S3***


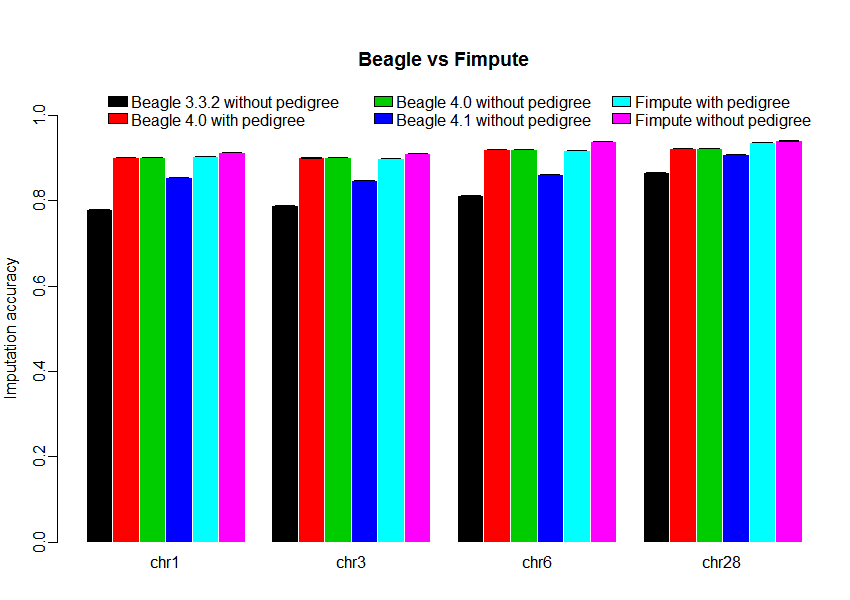


The average imputation accuracy of direct imputation from 600k to WGS data obtained with FImpute, Beagle v3.3.2, Beagle v4.0, and Beagle v4.1 for four chromosomes (chr1, chr3, chr6 and chr28) among 5 replications. The imputation accuracy were the genotype concordance between the true and imputed genotypes.
